# Artificial intelligence for predicting the risk of bone fragility fractures in osteoporosis

**DOI:** 10.1186/s41747-025-00572-3

**Published:** 2025-06-24

**Authors:** Fabio Massimo Ulivieri, Carmelo Messina, Francesco Maria Vitale, Luca Rinaudo, Enzo Grossi

**Affiliations:** 1 Bone Metabolic Unit, Rome American Hospital, Gruppo Nefrocenter, Roma, Italy; 2 U.O.C. Radiodiagnostica, ASST Centro Specialistico Ortopedico Traumatologico Gaetano Pini-CTO, Milan, Italy; 3 Dipartimento di Scienze Biomediche per la Salute, Università degli Studi di Milano, Milano, Italy; 4 IRCCS Istituto Ortopedico Galeazzi, 20161 Milan, Italy; 5 Tecnologie Avanzate T.A. s.r.l, Turin, Italy; 6 Fondazione Villa Santa Maria, Tavernerio, Italy

**Keywords:** Artificial intelligence, Bone density, Deep learning, Osteoporotic fractures, Osteoporosis

## Abstract

**Abstract:**

Osteoporosis is widespread with a high incidence rate, resulting in fragility fractures which are a major contributor to mortality among the elderly. Artificial intelligence (AI), in particular artificial neural networks, appears to be useful in managing osteoporosis complexity, where bone mineral density usually reduces with aging, losing the pivotal role in decision-making regarding fracture prediction and treatment choice. Nevertheless, only some osteoporotic patients develop fragility fractures, and treatments often are not prescribed because of the high costs and poor patient adherence. AI can help clinicians to identify patients prone to fragility fractures who can benefit from preventive interventions. We describe herein the methodology issues underlying the potential advantages of introducing AI methods to support clinical decision-making in osteoporosis, being aware of challenges regarding data availability and quality, model interpretability, integration into clinical workflows, and validation of predictive accuracy. The fact that no AI fracture risk prediction software is still publicly available can be related to the fact that few high-quality datasets are available and that AI models, particularly deep learning approaches, often act as ‘black boxes’, making it difficult to understand how predictions are made. In addition, the effective implementation of predictive software has not reached sufficient integration with existing systems.

**Relevance statement:**

With aging, bone mineral density may lose the pivotal role in osteoporosis decision-making regarding fracture prediction and treatment choice. In this scenario, AI, particularly artificial neural networks (ANNs), can be useful in supporting the clinical management of patients affected by osteoporosis.

**Key Points:**

- Osteoporosis is a complex disease with many interlinked clinical and radiological variables.
- Bone mineral density and other known indices do not allow optimal decision-making in patients affected by osteoporosis.
- ANN analysis can better discriminate osteoporotic patients particularly prone to fragility fractures and can predict future fractures.

**Graphical Abstract:**

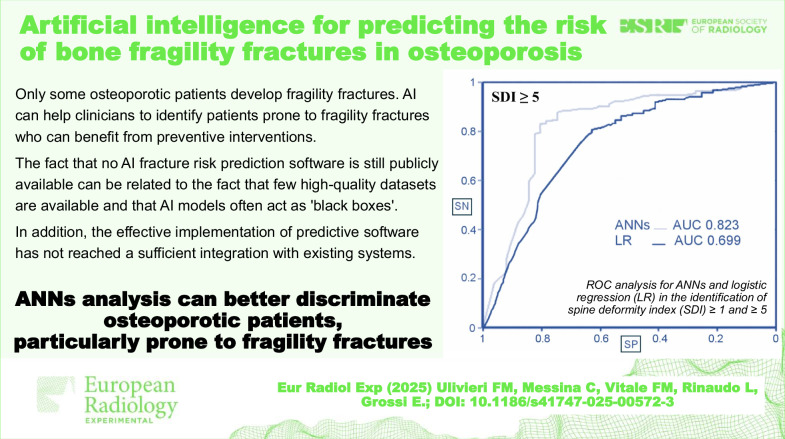

## Introduction

The recent development of artificial intelligence (AI) techniques has had a significant impact on the healthcare sector, fueling a debate about whether AI can replace physicians in various fields of medicine, including radiology [1].

While it is improbable that machines will replace physicians in the near future, it is beyond doubt that AI will help physicians, enhancing clinical decisions and validating human judgment, particularly in radiology. The growing accessibility of healthcare data and the swift progress in big data analysis methods facilitated the successful integration of AI in healthcare. Driven by pertinent clinical questions, robust AI techniques can uncover and emphasize clinically significant information within extensive datasets.

Artificial neural networks (ANNs) are adaptive AI systems that draw inspiration from the operational human brain processes [2, 3]. These data processing systems can adapt their internal structure based on the objectives of a function. Consequently, they are well suited for addressing nonlinear problems and can effectively reconstruct the fuzzy logic rules that govern the most favorable solutions [4, 5]. Adaptability to learn is a distinctive feature making ANN models a powerful data analysis tool. The organization of these systems can be adjusted in response to environmental changes, enabling ANNs to construct their unique representation of information. Additionally, such models exhibit notable noise tolerance, providing accurate performance even in the presence of unreliability, erroneous data, or measurement errors. The selection of a sophisticated inferential technique like ANNs is guided by the awareness that in those cases where the relationships among study variables are highly complex and there is no dependable *a priori* statistical model available, ANNs can be approached as an optimal solution. They demonstrate effectiveness in simultaneously managing a high number of variables, regardless of the essential degree of nonlinearity. This characteristic results in structurally robust outcomes, even when the underlying statistical process is not well understood [6].

With aging, bone strength decreases and the risk of fragility fracture increases [7]. The most important predictor of bone resistance is bone mineral density (BMD). However, although reduced BMD is the most frequent index for impaired bone, not all patients with reduced BMD experience fragility fracture, many other factors contribute to its occurrence [8]. The most important challenge in clinical practice is to identify those subgroups of patients with a higher risk of fracture, since treating all patients at risk of fracture indiscriminately with drugs for osteoporosis is neither feasible nor appropriate due to the need to balance drug efficacy, side effects, and healthcare costs [9]. Therefore, identifying high-risk subgroups is crucial to optimize treatment benefits while avoiding unnecessary therapies in patients with lower risk.

In this perspective, AI offers a useful contribution in identifying patients particularly prone to fragility fracture that may benefit most from pharmacological therapy [10–12].

In this review, we will consider these two fields of AI application: the fracture predictivity in the single individual and the disentanglement of disease complexity using a particular ANN method, the auto contractive map (autocontractive map (Auto-CM)). In addition

### AI-based fracture predictivity in a single individual

One of the most attractive properties of machine learning (ML) systems is the possibility of making an inference at a single individual level and not only a population level. The medical statements are based on evidence-based medicine criteria, which arose from population studies, but it is with the individual that the physician must relate to providing the best care and support. No two individuals are the same, and various degrees of uncertainty about diagnosis, treatment, or eventually, patient adherence to therapy have to be faced.

Most statistical methods currently in use for population studies analysis were developed during the last century’s first half, when medicine was primarily focused on acute infectious diseases with limited available clinical information. Today the doctor is confronted with a multitude of clinical data regarding the individual patient. Their role in the genesis of osteoporosis in an individual patient is not easily ponderable. In order to reduce fracture risk, it is not easily identified the key factor to focus attention on for the most appropriate diagnosis and therapy.

AI has been proposed as a potential assistant for this purpose. A recent systematic review with meta-analysis [10] on this argument highlighted the good accuracy of AI, still warning against the bias risk in patients’ selection and the high heterogeneity of the studies considered [13]. Traditional ML systems encompass many data analysis techniques designed to develop predictive models. These models learn from data and enhance their predictive capabilities through iterative experience gained from the data itself [14]. Before constructing an ML system, raw data must undergo preprocessing, which involves extracting and engineering features. This step necessitates domain expertise to ensure the algorithm is trained effectively [14].

Deep learning (DL) is an ML subfield that employs methods capable of learning intricate relationships between inputs and outputs. DL models stand out for their capability to handle raw data effectively, often reducing the need for extensive feature engineering. This is because they can model complex functions and autonomously identify relevant aspects within the data distribution. DL algorithms, based on ANNs, draw inspiration from the human brain structure. A convolutional neural network, an example of a DL algorithm, is structured into nodes organized within multiple layers. It can ingest an image as input, analyze its features across its layers, and generate an output that assigns a class label, effectively distinguishing between multiple groups or categories [14]. This is the case with auto-contractive maps.

Literature about ML applications in bone and mineral research has exponentially increased from 2015 to 2021, and in the last two years fracture detection and risk prediction represent 50% of the topics dealt [15]. A recent review of ML solutions for osteoporosis reveals that 15% of papers deal with fragility fracture detection and risk prediction. Hip fracture represents the primary goal of the studies, followed by other fractures [16]. Although the cited literature confirms the usefulness of AI and its subsets in osteoporosis, it emphasizes the risk of bias due to the technique’s nature and the data quality [15, 16].

As highlighted in a recent review by Smets et al [17], among the 32 studies that investigated fracture detection, 11 regarded vertebral fractures, 17 hip fractures, and 10 other fracture sites such as humerus or wrist. Nineteen studies developed DL models for image analysis [17].

The performance obtained with DL is better in comparison with shallow ML systems (*i.e*., those not using DL methods), with an average area under the receiver operating characteristic curve of 0.96 *versus* 0.87, respectively. Only two of 34 studies on supervised ML systems applied to osteoporosis diagnosis from data and images have been externally validated and applied to new cases prospectively enrolled [15]. External validation is essential for applying ML analyses in the real world as many studies on image-based radiologic diagnoses have shown reduced algorithm performance on external datasets [18]. In addition, quite shockingly, despite the abundance of studies using AI for fracture risk detection, no application software has yet to be publicly available for routine use.

AI has shown significant promise in predicting osteoporosis fractures through various methodologies and datasets. For instance, the Crystal Bone algorithm demonstrated an area under the receiver operating characteristic curve of 0.74–0.77 across three United States health systems, effectively identifying patients at risk of fractures within two years [19]. Additionally, an ML framework utilizing an open-source dataset achieved an impressive accuracy of 89% in predicting osteoporosis risk, employing techniques like the SHapley Additive exPlanations—SHAP for interpretability [20]. Furthermore, DL models, such as convolutional neural networks, have been reported to achieve up to 97.6% accuracy in detecting osteoporosis from x-ray images [21]. These advancements indicate that AI can enhance fracture prediction accuracy, particularly in populations with unique characteristics, such as diabetic patients [22]. However, while AI shows potential, its application in clinical settings requires further validation to ensure reliability and integration into existing healthcare frameworks.

**Fig. 1:**
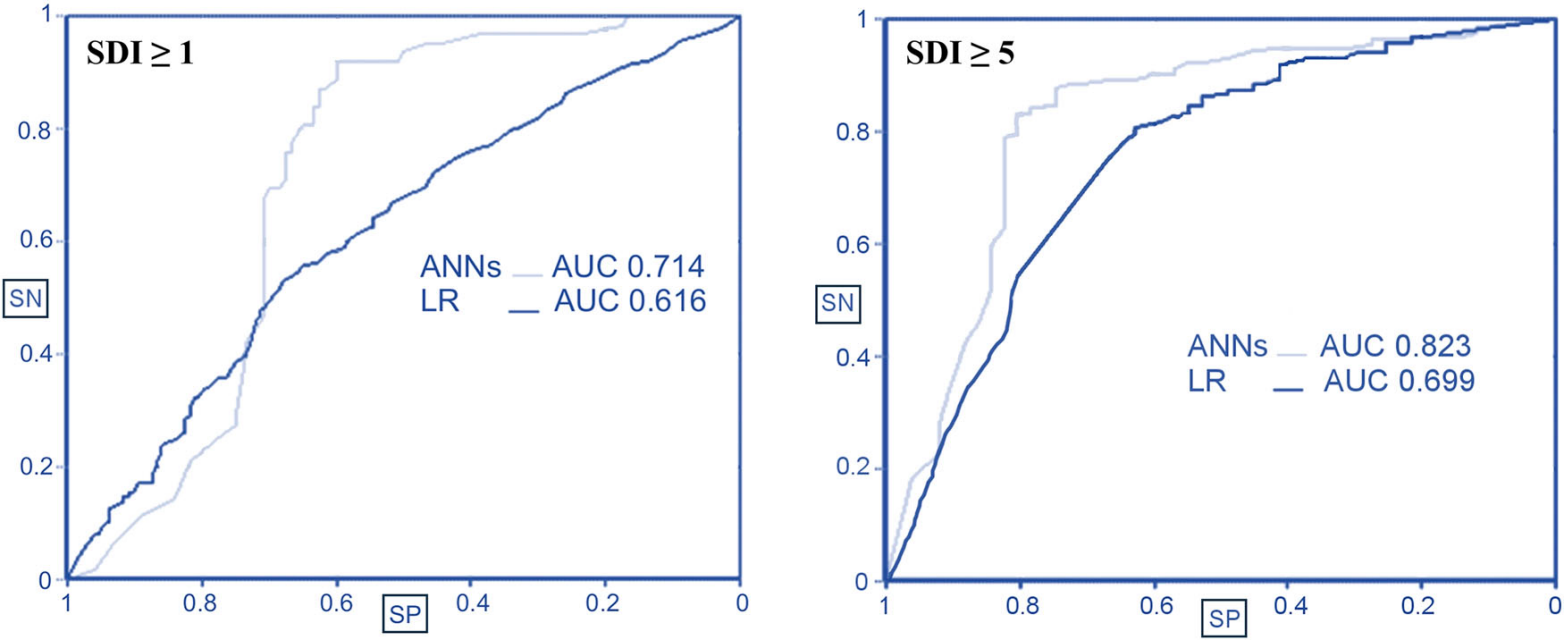
Receiver operating characteristic curves for ANNs and LR analysis in the identification of SDI ≥ 1 and ≥ 5. The ANN area under the curve (AUC) is superior to LR AUC in the identification of both SDI ≥ 1 (*p* < 0.01) and SDI ≥ 5 (*p* < 0.001). SN, Sensitivity; SP, Specificity. From: Eller-Vainicher et al [23], adapted with permission

According to Eller-Vainicher et al [23], supervised ANNs prognostic performance was compared with that of logistic regression (LR) in identifying vertebral fractures in 372 women suffering from postmenopausal osteoporosis [23]. BMD measured by dual-energy x-ray absorptiometry (DXA) only partially predicts the fracture risk, and the degree and number of vertebral fractures are strong predictors of developing future osteoporotic fractures. The spine deformity index (SDI), according to Genant classification [24], was used as a score that integrates both the number and the severity of morphometric vertebral fractures. One hundred and ninety-six women presented SDI = 0, 176 women SDI > 1, and 51 women SDI > 5. Besides, BMD was also considered in the analysis of 44 clinical parameters. ANNs can automatically select relevant input data using the training with input selection and testing (TWIST) Semeion system. Among a total of 45 variables, in the first analysis, the TWIST system selected 17 and 25 in SDI > 1 *versus* SDI = 0, while in the second analysis, the selection was made between SDI > 5 *versus* SDI = 0. In the first analysis, the sensitivity of LR and ANNs was 35.8% and 72.5%, the specificity was 76.5% and 78.5%, and the accuracy was 56.2% and 75.5%, respectively. Conversely, during the second analysis, the system showed a sensitivity of 37.3% (LR) and 74.8% (ANNs), specificity of 90.3% (LR) and 87.8% (ANNs), and accuracy of 63.8% (LR) and 81.3% (ANNs). The performance of ANNs was better in identifying both SDI > 1 and SDI > 5, demonstrating higher sensitivity, thus suggesting a relevant role of ANNs in developing valid algorithms for predicting osteoporotic fractures (Fig. 1).

### Disentanglement of osteoporosis complexity with AI data mining

**Fig. 2:**
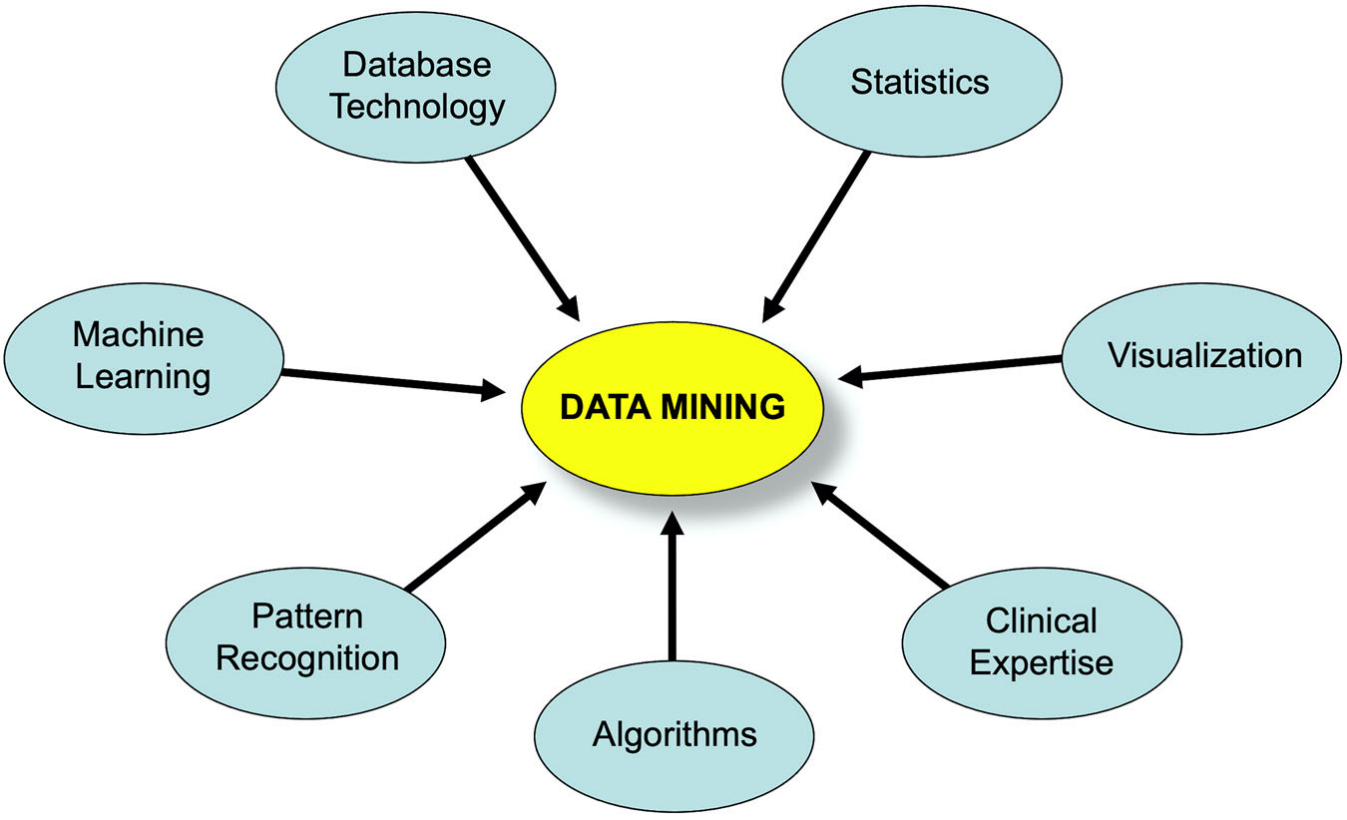
Disciplines connected with data mining

Data mining involves extracting exciting patterns or knowledge from large datasets. These patterns are often implicit, previously unknown, and potentially beneficial. Data mining is the confluence of multiple disciplines, as described in Fig. 2.

Data mining has been commonly used to extract useful information from business data [25, 26]. Still, it has emerged as a valuable tool also in medical applications, as a modern approach that has emerged alongside the rise of genomic and functional data. Data mining extends beyond conventional data analysis by integrating traditional tools for analysis like statistics or graphics with AI techniques, such as neural networks and rule induction. Data mining is becoming popular in medicine because efficient tools are needed to uncover valuable and previously unknown information from health-related data sources. Many variables have weak and mathematically loose relationships when dealing with chronic, complex diseases. For this reason, it is not easy to represent the general pattern with standard statistical tools. In other words, the emergent relationships among the variables may represent the actual structure of that specific medical disorder for which the variables were collected rather than the variables themselves [27].

The data mining techniques provided by classical statistics, such as principal component analysis and hierarchical clustering, face limitations due to the intricate interactions between risk factors, their nonlinear effects on disease occurrence, and significant stochastic components [12]. The most commonly used algorithms for linear plotting variables typically assume a Gaussian data distribution and exhibit limited efficacy when dealing with non-linear relationships between variables. Moreover, these algorithms often fail to preserve the geometric structure of the original data space. Applying these methods can result in losing important information, particularly when establishing precise associations among variables with only contiguity as a known element, which can be problematic.

Another limitation of current statistical methods is their reliance on specific types of “distance” metrics among variables (such as City block, Euclidean, correlation, etc.), which result in a “static” projection of potential associations. This approach needs to capture the intrinsic dynamics arising from active interactions among variables in real-world living systems, which could be effectively captured by ML systems [12]. Traditionally, many data mining algorithms rely on clustering variables. Clustering involves grouping similar data objects based on a similarity measure. In most applications, the similarity measure used in clustering is based on distance functions such as Euclidean distance, Manhattan distance, Minkowski distance, and Cosine similarity, among others. Clusters are formed so that data objects within the same cluster are minimally distant from each other, while data objects in different clusters are maximally distant from each other [28].

With traditional clustering techniques, several problems arise that are not easily solved: for example, it is necessary to determine a priori the number of the existing clusters, and the same variable may belong to two clusters simultaneously, depending on this numerical choice. It is also impossible to assign a hierarchy of importance to individual components, *i.e*., which of them commands the system. Finally, label visualization is often difficult in the case of multiple variables. These limitations are largely overcome with the use of the minimum spanning tree (MST) method.

### A conceptual description of the MST

**Fig. 3:**
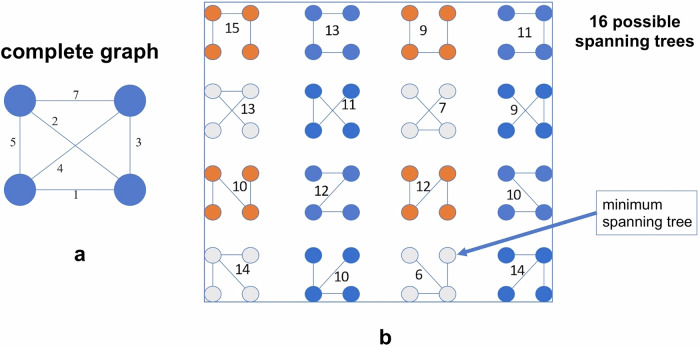
Example of an MST graph. The figure has two panels. The left side, **a** shows a fully connected graph where every point is linked to all others (with Arabic numbers for the corresponding distance). On the right, **b** illustrates the 16 possible spanning trees connecting the four points without forming loops. among these, the MST is the one that connects all points using the shortest total distance (sum = 6)

An MST is a connected, undirected graph is a tree that links all vertices together with the least total weight possible for its edges. Numerous distinct spanning trees can be associated with a single graph. It is possible to assign a weight to each edge, which represents its “cost” or “unfavorability” and use these weights to calculate the total weight of a spanning tree. This is done by summing the weights of the spanning tree’s edges. An MST is defined as a spanning tree with a weight that is less than or equal to every other possible spanning tree of the same graph. In Fig. 3, the theory applied to 4 points or variables is graphically shown, where the distances between them are represented on the arches within a multidimensional space: panel a shows a complete graph where all points are interconnected; panel b illustrates 16 potential spanning trees, which are ways to connect the four points without creating loops. Given the distances between points, there is one spanning tree where the total distance is minimized (sum = 6), representing the MST for this set of points.

There are several practical applications for MSTs. For example, a telecommunications company might use an MST to efficiently lay out cables in a new neighborhood, ensuring all locations relate to the minimum total cable length. There may be several possible spanning trees, each representing a different way to achieve full connectivity without forming any loops. Regarding the MST, this may be represented by the one with the lowest total cost, representing the least expensive configuration for laying the cable while connecting all points without forming any cycles. MST was developed specifically for this purpose [29–31].

The MST filter is applicable in the biomedical field, notably in microarray clustering. While MST-based clustering is formally equivalent to dendrograms generated by hierarchical clustering under specific conditions, visually, their outputs can differ significantly [14]. MST provides the optimal method for connecting variables in a tree structure, ensuring the shortest possible connections that simplify the presentation of data in a graphical form. The assumption is rooted in the idea that biological systems tend to adopt states of minimal energy. This graph represents the fundamental biological information of the system, encapsulating this principle. The primary objective of this model is to uncover hidden trends and explore non-linear associations among variables, particularly in central hubs. Hubs are typically defined as variables with the maximum number of connections on the map [30].

In biological systems, the paths derived from the principle of least action quantify the kinetic transitions between normal and pathological states. Therefore, we assume that in the case of variables describing normal and pathological states, their interconnected system naturally tends toward minimal length, a concept well represented by the graph generated by MST. Practically speaking, MST demonstrates the optimal method to connect variables in a tree, achieving the shortest possible connections and presenting data in a simplified graph format.

The main advantage of the MST algorithm is its ability to provide a concise overview of the ensemble of variables, making clustering easy to understand by directly linking closely related variables. The significance of variables in the graph correlates with their number of links. The clustering distance between two variables is determined by their degree of separation. Among the different possible similarity functions used to obtain a distance matrix to be filtered by MST, we describe here a distance matrix generated by the weights of a fourth-generation artificial neural network called AUTO-CM.

### The Auto-CM ANN

The Auto-CM system is a fourth-generation unsupervised ANN with a three-layer architecture. It computes the multidimensional associations of the strength of each variable with all others in a dataset using a mathematical approach grounded in recursive non-linear equations [32]. The three Auto-CM layers are:

1. An input layer, captures signals from the environment.
2. A hidden layer, where these signals undergo modulation within the Auto-CM.
3. An output layer, through which the Auto-CM influences the environment based on the stimuli it has previously received.

Buscema and Grossi developed, tested, and implemented the architecture and mathematics of Auto-CMs in the C language in 1999 at the Semeion Research Center of Sciences of Communication in Rome [33]. After a training phase, the Auto-CM determines the “weights” of the vectors matrix, which reflect the strength of many-to-many associations among all variables. These weights can be visualized by transforming them into physical distances: variables with higher connection weights are positioned closer to each other, while those with lower weights are relatively farther apart. This visualization helps in understanding the relationships and clustering structure of the variables based on their associations in the dataset.

The Auto-CM algorithm has a specificity to minimize a complex cost function

$$E = \,	 Min \left\{{\sum }_{i}^{N}{\sum }_{j}^{N}{\sum }_{k}^{N}{\sum }_{q}^{M}{u}_{i}^{q}\cdot {u}_{j}^{q}\cdot {u}_{k}^{q}\cdot {A}_{i,j}\cdot {A}_{i,k} \right\}; \\ {{{\bf{A}}}} = \,	 (1.0-\frac{{{{\bf{w}}}}}{C}); \\ N = \,	 {{{\rm{Number}}}}\,{{{\rm{of}}}}\,{{{\rm{Variables}}}}\,({{{\rm{Columns}}}}); \\ M = \,	 {{{\rm{Number}}}}\,{{{\rm{of}}}}\,{{{\rm{Patterns}}}}\,({{{\rm{Rows}}}}).$$

At this stage, the dataset is converted into an undirected weighted graph, allowing the application of the MST algorithm.

We evaluated MST coupled with Auto-CM in different papers to develop an individual model to predict the possible future incidence of vertebral fractures. This was specifically done, for example, to evaluate the contribution of bone strain index (BSI), a DXA metric of bone deformation under loads, to predict fragility fracture, offering further insight regarding the complex biological relationship among several osteoporotic variables and the two conditions (fracture *versus* not-fracture). BSI is a DXA-based tool that integrates BMD with the mechanical behavior of bone under stress using a finite element analysis of DXA images.

**Fig. 4:**
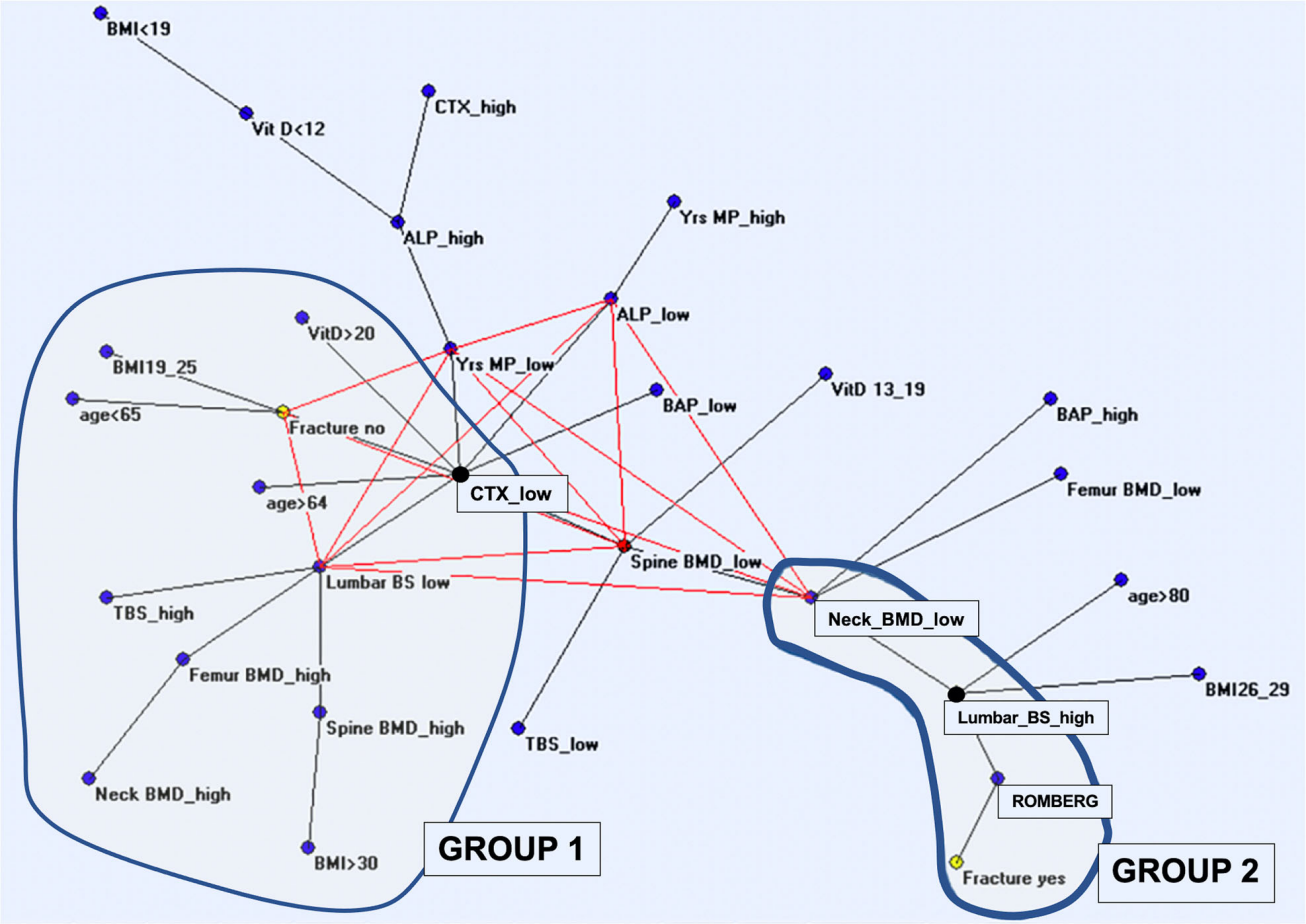
Minimal spanning tree and the maximally regular graph of the interrelations between DXA bone status parameters, balance conditions, and fractures. From Ulivieri et al [10], adapted with permission

The use of BSI together with BMD has been showed to further refine fracture risk assessment [34]. Ulivieri et al [10] applied MST-AutoCM to investigate bone status (bone quantity and bone quality) in 125 postmenopausal women using DXA BMD, BSI, and biochemical markers of bone turnover, morphometric vertebral fractures and balance condition (Romberg test) [10]. The resulting semantic map (Fig. 4) depicts the “maximally regular graph” and the MST of the interrelations among DXA bone status parameters, balance conditions, and fracture data for the studied population. Low bone marker levels correlate with a reduced fracture risk. Conversely, a positive Romberg test and decreased bone tensile resistance to loads (measured with BSI) are strongly associated with fragility fractures [10].

In particular, the maximally regular graph identifies two postmenopausal women subgroups. In the first group, the marker of bone resorption “C-telopeptide low”, suggesting an insufficient bone remodeling status, seems to be the central hub for numerous connections involving most variables related to the bone status, such as bone mineral metabolism, bone quantity, and quality, patient characteristics, and fracture status (“Fracture no”). On the contrary, the second group, is characterized by a scanty number of bone status indexes involved, with very rarefied connections, typical of a non-normal biological system, involving a very poor state of bone quality measured with Bone Strain (“BS high”) and a propensity to fall (Romberg test positive). This work is paradigmatic of how the ANNs approach appears to be suitable for analyzing complex clinical systems with many variables of different significance, like postmenopausal osteoporosis, suggesting how a simple approach enables clinicians to identify frail patients at risk of fractures who would benefit most from prevention treatments.

**Fig. 5:**
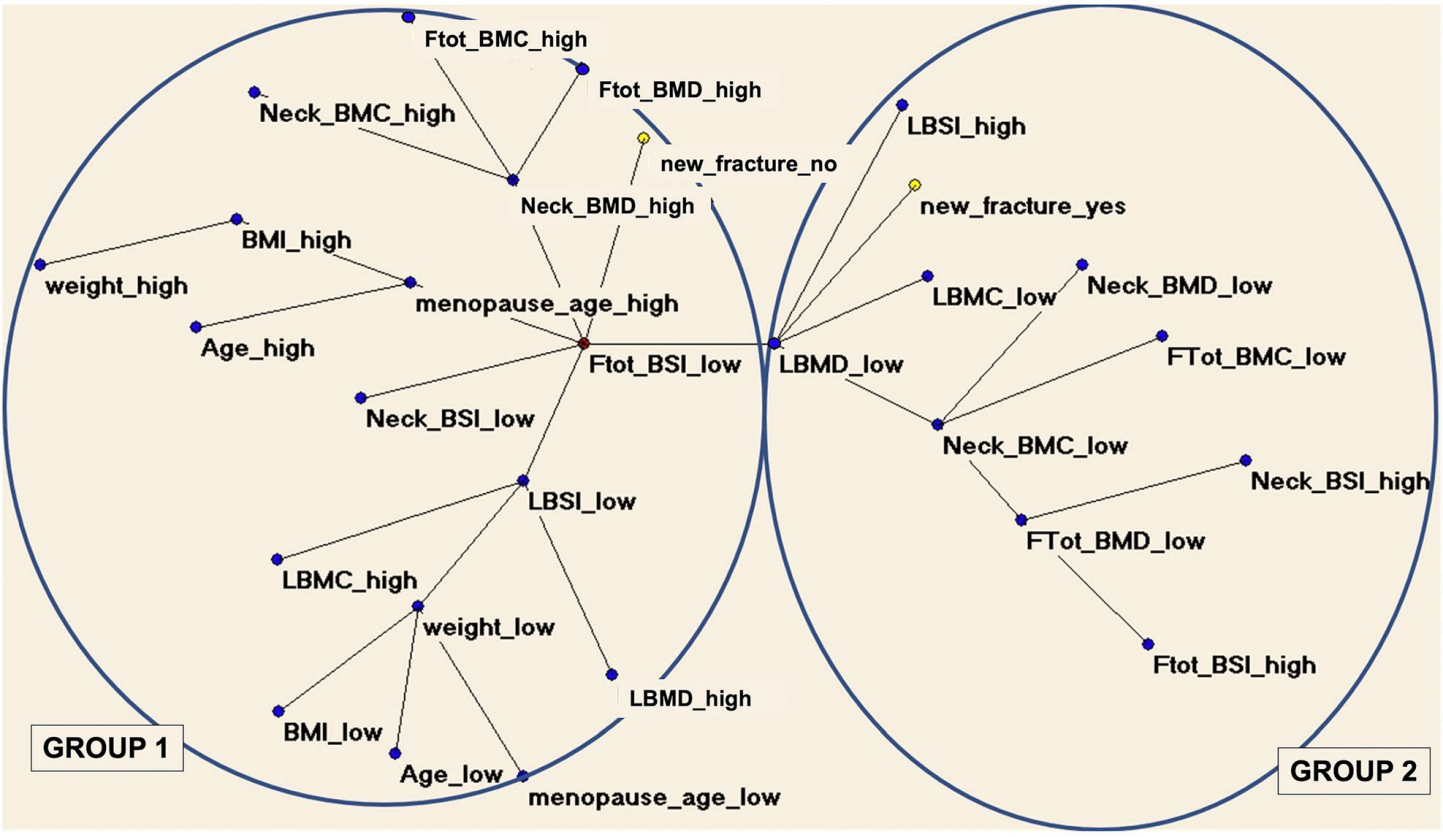
Semantic connectivity map shows the connections among many parameters, including those DXA-based. The map identifies two groups: group 1, with low BSI (a DXA-based parameter of bone resistance to load) at the total femur (Ftot_BSI_low) being associated with the absence of vertebral fractures; group 2, with low values of lumbar bone mineral density (LBMD). BSI, Bone strain index; DXA, Dual-energy x-ray absorptiometry; BMC, Bone mineral content; BMI, Body mass index; Ftot, Total femur; L”, Lumbar; “low”, Low values of the specific parameter; “high”, High values. From Ulivieri et al [32], adapted with permission

Ulivieri et al [32] employed ANNs to forecast the fragility of vertebral fractures in postmenopausal women [32]. They used DXA parameters in a cohort of 174 postmenopausal women who were initially free of vertebral fractures. Based on the occurrence of new vertebral fractures during follow-up, two groups were distinguished: those who experienced fractures (fractured group) and those who did not (non-fractured group). The authors used ANN analysis (MST-AutoCM) in conjunction with an optimizing tool (TWIST system) to identify important input data from a set of 13 variables. This set included measurements such as BMD and BSI, assessed at both vertebral and femoral sites. At follow-up, 69 women (39.6%) developed a vertebral fracture. The ANNs achieved a predictive accuracy of 79.6% during the training and testing procedure, with a sensitivity of 80.9% and a specificity of 78.2%. According to the semantic connectivity map, low femoral BSI (meaning a good resistance to loads) was associated with the absence of vertebral fractures (group 1), showing high performance in detecting the unlikeliness of vertebral fractures (Fig. 5). This is relevant because it shows that a bone densitometry variable measured at the femur is helpful to foresee a low probability of fracture of another skeletal site, lumbar spine.

A similar multicentric longitudinal study was performed on 172 female subjects with at least one vertebral fracture at the first observation [11]. Subjects were evaluated using the SDI plus DXA scans to collect data about BMD and BSI at baseline and after a follow-up period of about 3 years. By the conclusion of the follow-up period, 93 women experienced an additional vertebral fracture. MST-AutoCM appropriately chose the data input automatically, and the TWIST system differentiated between women who experienced an additional fracture and those who did not, identifying the variables that provided the most significant information to distinguish between the two groups. Auto-CM offered further insights into the complex relationships among osteoporotic variables considered, highlighting their connections in both scenarios: those with a further fracture and those without. In particular, further fracture event was strictly connected to lumbar spine high BSI, indicating a reduced capability of bone to deform under loads. On the contrary, the absence of further fracture events is associated with a low lumbar spine BSI.

**Fig. 6:**
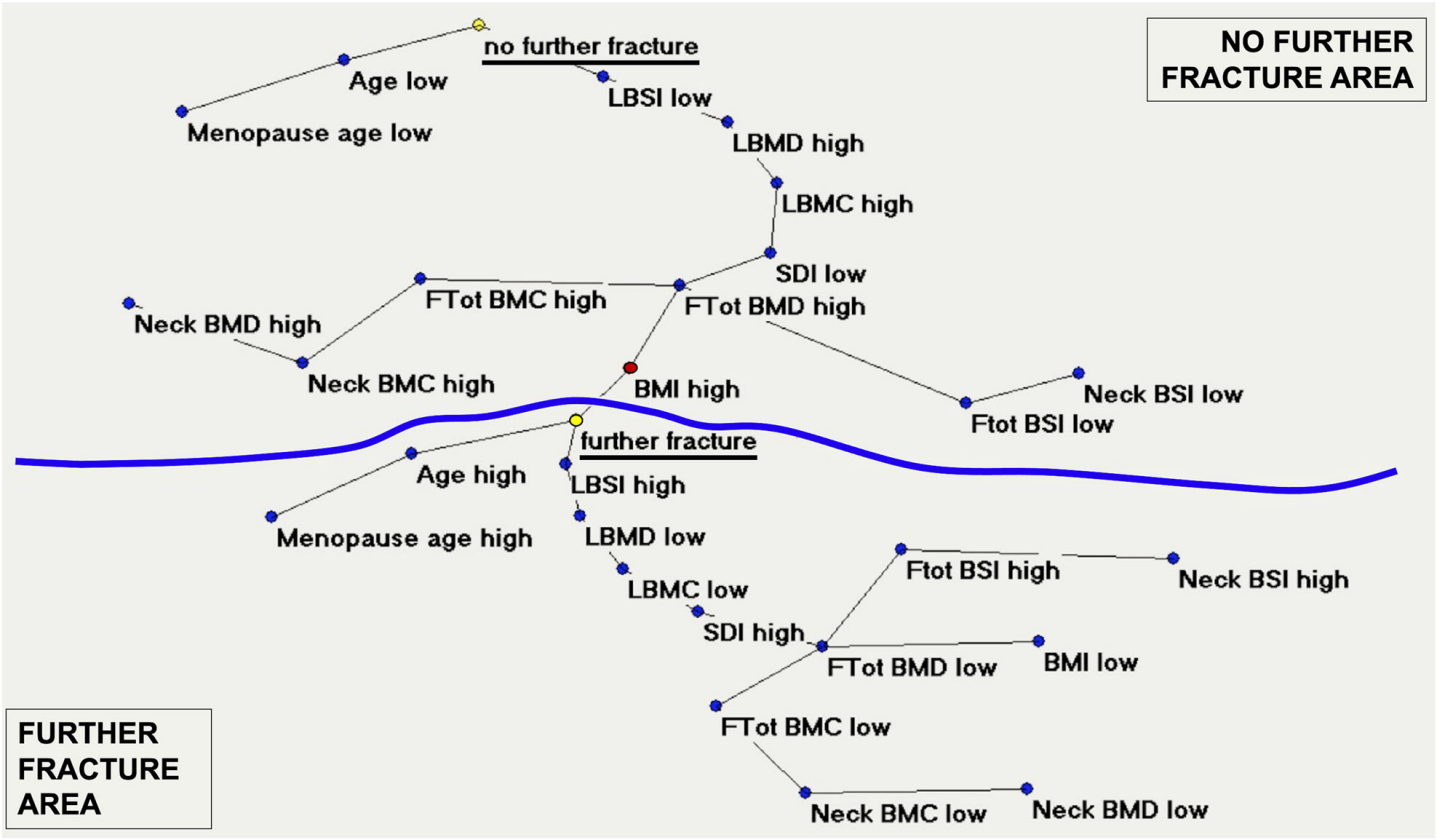
Semantic connectivity map with a maximally regular graph showing the two separate areas (further fracture *versus* no further fracture), with the clinical and DXA-based variables involved. BSI, Bone strain index; DXA, Dual-energy x-ray absorptiometry; BMC, Bone mineral content; BMI, Body mass index; Ftot, Total femur; “L”, Lumbar; “low”, Low values of the specific parameter; “high”, High values. From Ulivieri et al [11], adapted with permission

Only another variable, age, is so strictly near to the event of further fracture, with the same behavior of BSI (Fig. 6). DXA variables, usually considered the most important for predicting further fragility fractures, resulted in the neural map being less important than the index of the ability to deform under loads of the bones. These physical aspects, the deformation capability of the material under loads, appeared to be more significant than the material density in the pathogenesis of further fragility fractures. In this study, ANNs achieved a predictive accuracy of 79.4%, demonstrating a sensitivity of 75.0% and a specificity of 83.7%. The semantic connectivity map underscored the significance of BSI in predicting the risk of subsequent fractures, suggesting AI as a valuable method for analyzing complex systems such as osteoporosis. The possibility to use an index (BSI) that enhances the identification of patients at greater risk of further fragility fractures is of clinical relevance in choosing the best drug therapy for secondary prevention in osteoporosis.

**Fig. 7:**
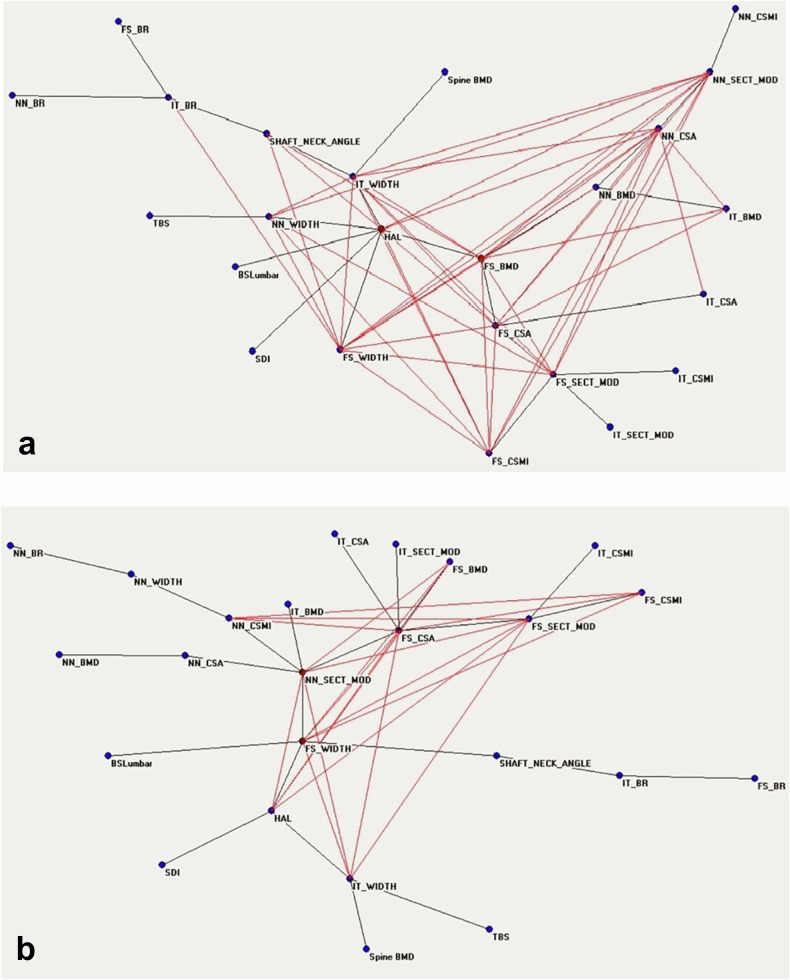
Semantic map showing the relations between demographic, densitometric, biochemical, and anthropometric before (**a**) and after (**b**) treatment with teriparatide. BMD, Bone mineral density; BR, Buckling ratio; BSI, Bone strain index; CSA, Cross-sectional area; CSMI, Cross-sectional moment of inertia; SEC_MOD, Section modulus; FS, Femoral shaft; HAL, Hip axis length; IT, Intertrochanter; NN, Narrow neck; TBS, Trabecular bone score. Adapted from Messina et al [12]

Messina et al [12] used ANNs mentioned above to indagate the capability of this AI method to identify the response to pharmacological treatment of osteoporosis able to improve both bone quantity and bone quality (evaluated with DXA as BSI, trabecular bone score, and bone geometry parameters) [12]. Patients were subdivided by BMD increase, assumed as an *a priori* condition according to the registration study of Neer et al [35]. MST-AutoCM revealed that bone quality and bone geometry parameters changed their position and importance in the connections’ semantic map after treatment (Fig. 7).

**Fig. 8:**
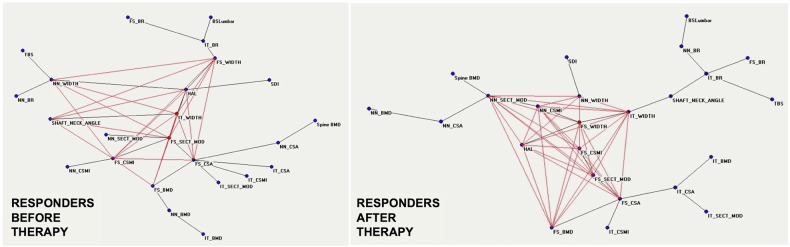
Semantic maps showing the relations between clinical, laboratory, and DXA-based parameters of the responder's group before treatment and after the treatment. BMD, Bone mineral density; BR, Buckling ratio; BSI, Bone strain index; CSA, Cross-sectional area; CSMI, Cross-sectional moment of inertia; SEC_MOD, Section modulus; FS, Femoral shaft; HAL, Hip axis length; IT, Intertrochanter; NN, Narrow neck; TBS, Trabecular bone score. Adapted from Messina et al [12]

**Fig. 9:**
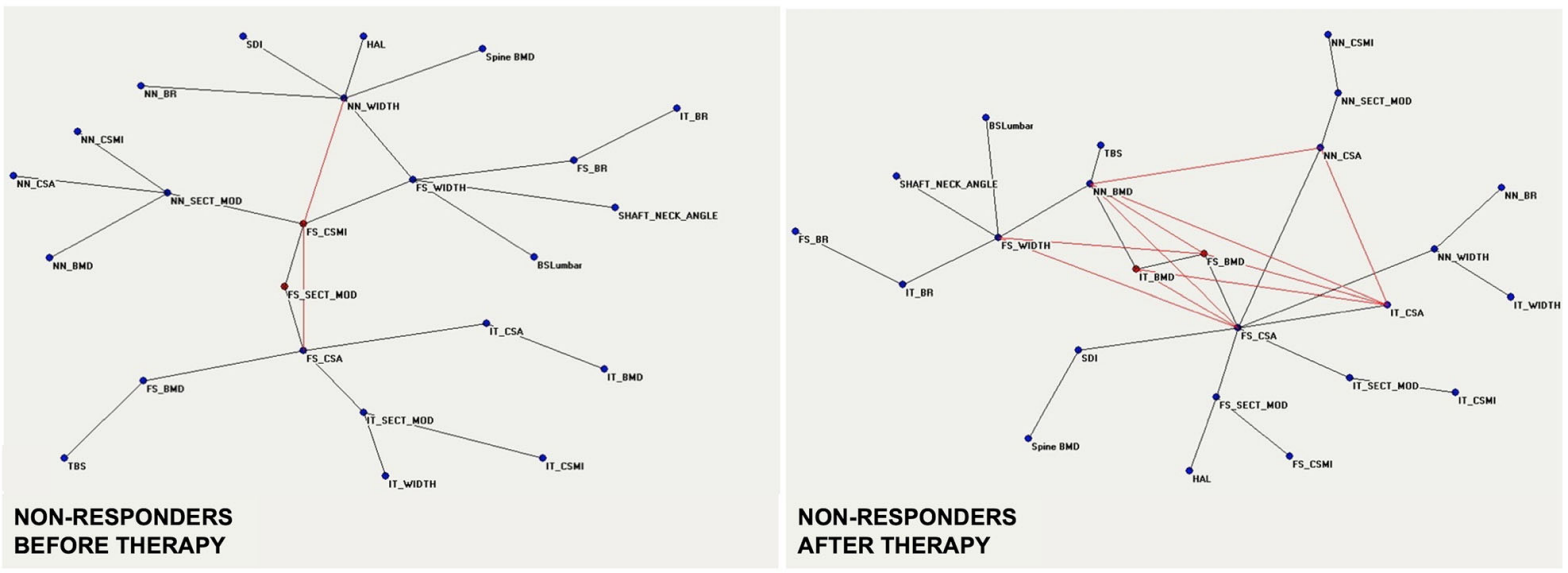
Semantic maps showing the relations between clinical, laboratory, and DXA-based parameters in the non-responders group before treatment and after the treatment. BMD, Bone mineral density; BR, Buckling ratio; BSI, Bone strain index; CSA, Cross-sectional area; CSMI, Cross-sectional moment of inertia; SEC_MOD, Section modulus; FS, Femoral shaft; HAL, Hip axis length; IT, Intertrochanter; NN, Narrow neck; TBS, Trabecular bone score. Adapted from Messina et al [12]

Before anabolic treatment, there is a notable distribution centered around two key nodes: “FS_BMD,” which represents the femoral axial bone density index, and “HAL,” indicating hip axis length. These nodes are central hubs in the network, as illustrated in Fig. 7a. On the contrary, the distribution of nodes and their connections after therapy presents the “NN_SECT_MOD” index correlated with bending and torsion resistance, a central position of the connections’ map (Fig. 7b). This highlights the importance of anabolic treatment, teriparatide, in geometric bone quality variables changes, an aspect that clinicians consider insufficiently with respect to bone quantity, *i.e*., BMD variations. Still, the paradigmatic example of the usefulness of AI in medicine is the identification of a prediction model that allows clinicians to know the tendency to non-response before starting treatment (Figs. 8 and 9). When considering the two groups, responders and nonresponders to treatment, there were noticeable differences in the behavior of DXA variables. The ANNs maps of responders before and after treatment (Fig. 8) present increased connections between variables after anabolic treatment, and the geometric DXA variable “FS_CSMI”, index related to the strength to compressive, flexural, and torsional loads, becomes the central hub of the interconnections. The maps of “non-responders” before and after treatment (Fig. 9) reveal a significant lack of interconnections. After treatment, the variable “FS_CSMI” loses its central hub position to “FS_CSA,” an index more closely associated with femoral axial cross-sectional strength. Comparing the maps before treatment of responders and non-responders, the difference in bone quality status of the two groups appears evident and allows doctors to consider the therapeutic choice with a major appropriateness compared to the clinical data usually considered today.

### Limitations and conclusions

Developing AI-based fracture risk prediction tools in osteoporosis faces several challenges. These include data availability and quality, model interpretability, integration into clinical workflows, and validation of predictive accuracy. The fact that no fracture risk prediction software is publicly available can be related to the fact that few high-quality datasets are available and that AI models, particularly DL approaches, often act as ‘black boxes’, making it difficult for clinicians to understand how predictions are made. Another reason could be that the effective implementation of predictive software has not reached a sufficient level of seamless integration with existing systems.

We are aware of several limitations of AI in the osteoporosis field. For example, many AI models rely on high-quality datasets; however, osteoporosis data can be sparse or inconsistent, affecting model training and performance. In addition, the reliance on DXA scans for data collection can be problematic, especially in developing countries where access is limited.

To summarize, AI, particularly ANNs such as MST-AutoCM, appears to be useful in managing osteoporosis complexity, a disease where BMD usually reduces with aging, losing the pivotal role in osteoporosis decision-making regarding fracture prediction and treatment choice. Osteoporosis is widespread and has a high incidence rate. Fragility fractures, which result from osteoporosis, are a major contributor to mortality among the elderly [7]. Nevertheless, only some osteoporotic patients develop fragility fractures, and treatments often are not prescribed because of the high costs and the poor adherence of patients. AI can help clinicians to identify the subsets particularly prone to fragility fractures and that can better benefit from preventive health interventions.

## Data Availability

Not applicable.

## Abbreviations

AI: Artificial intelligence
ANN: Artificial neural networks
Auto-CM: Autocontractive map
BMD: Bone mineral density
BSI: Bone strain index
DL: Deep learning
DXA: Dual-energy x-ray absorptiometry
LR: Logistic regression
ML: Machine learning
MST: Minimum spanning tree
SDI: Spine deformity index
TWIST: Training with input selection and testing
